# Interaction of *Clostridium perfringens* Iota Toxin and Lipolysis-Stimulated Lipoprotein Receptor (LSR)

**DOI:** 10.3390/toxins10100405

**Published:** 2018-10-08

**Authors:** Masahiro Nagahama, Masaya Takehara, Keiko Kobayashi

**Affiliations:** Department of Microbiology, Faculty of Pharmaceutical Sciences, Tokushima Bunri University, Yamashiro-cho, Tokushima 770-8514, Japan; mtakehara@ph.bunri-u.ac.jp (M.T.); kobakei@ph.bunri-u.ac.jp (K.K.)

**Keywords:** *C. perfringens* iota toxin, lipolysis-stimulated lipoprotein receptor (LSR), internalization

## Abstract

Iota toxin produced by *Clostridium perfringens* is a binary, actin ADP-ribosylating toxin that is organized into the enzymatically active component Ia and the binding component Ib. Lipolysis-stimulated lipoprotein receptor (LSR) has been identified as a cellular receptor of Ib. Here, we investigated the functional interaction between Ib and LSR, where siRNA for LSR blocked the toxin-mediated cytotoxicity and the binding of Ib. The addition of Ib to LSR-green fluorescence protein (GFP)-transfected cells at 4 °C resulted in colocalization with LSR and Ib on the cell surface. Upon transfer of the cells from 4 °C to 37 °C, LSR and Ib were internalized and observed in cytoplasmic vesicles. When the cells were incubated with Ib at 37 °C and fractionated using the Triton-insoluble membrane, Ib oligomer was localized in insoluble factions that fulfilled the criteria of lipid rafts, and LSR was clustered in lipid rafts. To examine the interaction between N-terminal extracellular region of LSR and Ib, we constructed a series of LSR N-terminal deletions. Ten amino acids residues can be deleted from this end without any reduction of Ib binding. However, deletion of 15 N-terminal residues drastically reduces its ability to bind Ib. These results demonstrate that Ib binds to the LSR N-terminal 10 to 15 residues and endocytoses into trafficking endosomes together with LSR.

## 1. Introduction

*Clostridium perfringens* iota toxin consists of two distinct protein components, one being the cell-binding protein (Ib) and other the enzymatic protein (Ia) [1,2,3,4,5,6]. Ia possesses actin-ADP-ribosyltransferase activity, blocking the formation of F-actin in the cytosol and then disrupting the actin cytoskeleton [2,6,7,8]. It has been reported that lipolysis-stimulated lipoprotein receptor (LSR) is a cellular receptor for Ib, promoting Ia internalization into a target cell [6,9]. Iota toxin is a clostridial binary actin-ADP-ribosylating toxin, which family encompasses *Clostridium botulinum* C2 toxin (C2I and C2II), *Clostridium difficile* transferase (CDTa and CDTb), and *Clostridium spiroforme* toxin (CSTa and CSTb) [1,2,3,4].

Iota toxin is taken up into host cells by endocytosis, inducing cell rounding by using endosomal transport [3,4,5,6]. Ib binds to LSR on the host cell surface and forms an oligomer [6,9]. After the association of Ia with Ib oligomer, the toxin complex is internalized [3,4,5,6]. In an acidic early endosome, Ia is transported into the cytoplasm via pores in the early endosomal membrane formed by Ib. Ib is then trafficked from early endosomes to recycling endosomes and late endosomes [6,10,11,12]. For a long time, it has been understood that the individual components of the toxin lack toxic activity, but the combination of components exhibits cytotoxic, lethal and dermonecrotic effects [1,2,3]. On the other hand, we have previously reported that Ib only induces cell injury in A549 and A431 cells [13]. Ib caused severe ATP depletion in the sensitive cells accompanying cell swelling. Ultrastructural analysis confirmed rapid cell necrosis of Ib-treated cells. These data demonstrated that Ib induces cellular necrosis of A549 and A431 cells [13]. Recently, we found that acid sphingomyelinase (ASMase) derived from Ib-induced lysosome exocytosis facilitates the internalization of iota toxin [14].

Iota toxin binds and internalizes host cells through LSR [6,9,15]. LSR is a type I single-pass transmembrane proteinaceous receptor that plays a role in the organization of the tricellular tight junction [16,17]. It contains an extracellular N-terminal Ig-like domain, a single transmembrane region, and a long C-terminal cytoplasmic tail [16,17]. LSR was first discovered as a receptor for triglyceride-rich and low-density lipoproteins [18]. Furthermore, it has been reported that LSR plays a critical role in the formation of tricellular tight junctions that are involved in epithelial barrier functions [16,17]. Tricellulin and LSR are two principal protein constituents of tricellular tight junctions [19,20], where tricellulin forms a barrier to macromolecules in tricellular tight junctions [21]. LSR recruits tricellulin to tricellular tight junctions, indicating that the LSR and tricellulin play an important role in the barrier function [17]. In epithelial cells, LSR is responsible for constituting the critical tricellular tight junction barrier in combination with tricellulin [19,22]. Recently, it has been reported that the C-terminal domain of Ib comprising Ib amino acid residues 421 to 664 (Ib421–664) binds in LSR-expressing cells [23]. Ib421–664 binding results in a profound decrease of LSR and tricellulin from the tricellular tight junction, which promotes the passage of macromolecular solutes [23,24]. Ib421–664 modulates the permeabilization of large molecules via a paracellular pathway, by destroying tricellulin recruitment at tricellular tight junctions [23,24].

Iota toxin internalizes into target cells through receptor-mediated endocytosis [1,2,3,4,5,6]. Ib selectively binds to LSR on the cell surface of target cells through Ib421–664 and translocates to cell membrane lipid rafts [6,11]. However, it remains unclear how LSR contributes to Ib internalization. Here, we examined the functional interaction between Ib and LSR during iota toxin endocytosis.

## 2. Results

### 2.1. Effect of LSR siRNA on Iota Toxin-Induced Cytotoxicity

**Figure 1:**
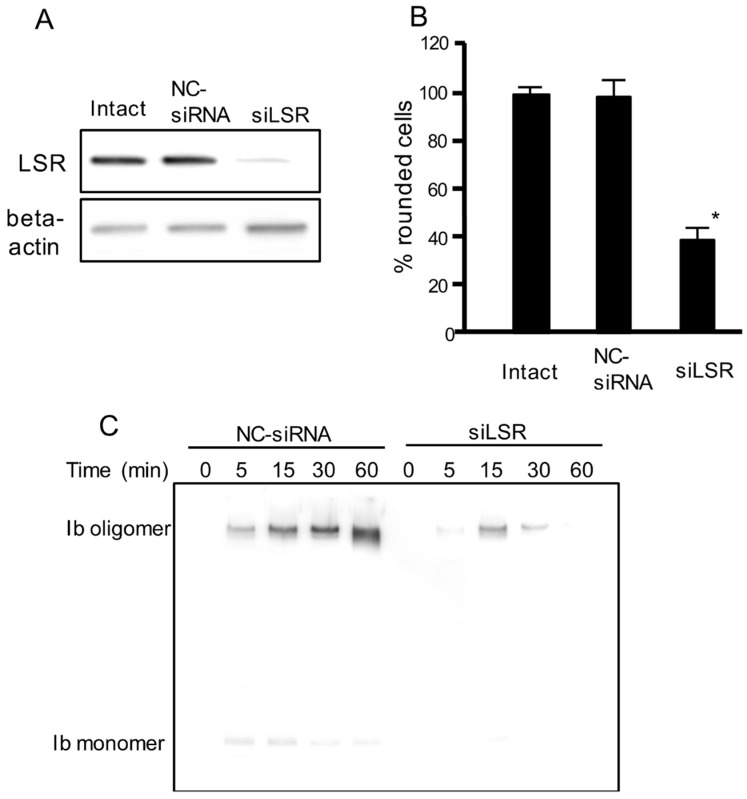
Effect of siRNA for the LSR on cell rounding activity and binding of iota toxin in Madin-Darby canine kidney (MDCK) cells. Short interfering RNAs (siRNAs) were utilized to decrease lipolysis-stimulated lipoprotein receptor LSR (siLSR) with a non-silencing siRNA used as control (negative control (NC)-siRNA). (**A**) Western blotting was utilized to assess the level of LSR knockdown. A typical example from one of three experimental studies is shown; (**B**) siRNA treated-cells or intact cells were incubated with Ia (200 ng/mL) and Ib (400 ng/mL) for 4 h at 37 °C. About 200 cells were counted on the microscopic photographs and the rounded cell numbers were expressed as percentages. Values are given as the mean ± standard deviation (SD) (*n* = 4). Significance was tested by two-tailed Student’s t-test (* *p* < 0.01, as compared to NC-siRNA-treated cells plus iota toxin); (**C**) These siRNA treated-cells were incubated with Ib (500 ng/mL) for 30 min at 37 °C. After washing, cells were then lysed, and Ib was detected by Western blotting using anti-Ib antibody. A typical example from one of three experimental studies is shown.

To examine the role of LSR in iota toxin-mediated cytotoxic activity, we performed an RNA interference (RNAi)-mediated knockdown of LSR. As shown in Figure 1A, LSR siRNA-treated cells showed decreased LSR expression. LSR knockdown markedly inhibited the iota toxin-induced cytotoxic activity compared with intact cells or negative control (NC)-siRNA-treated cells (Figure 1B). Next, we investigated the binding and oligomerization of Ib on NC-siRNA-treated or LSR-siRNA-treated cells. After incubation of NC-siRNA-treated cells with Ib, Ib oligomer was observed (Figure 1C). However, the level of Ib oligomer was greatly reduced in LSR-siRNA-treated cells. This confirmed that LSR was required for the cellular reception of Ib.

### 2.2. Internalization of Ib and LSR

**Figure 2:**
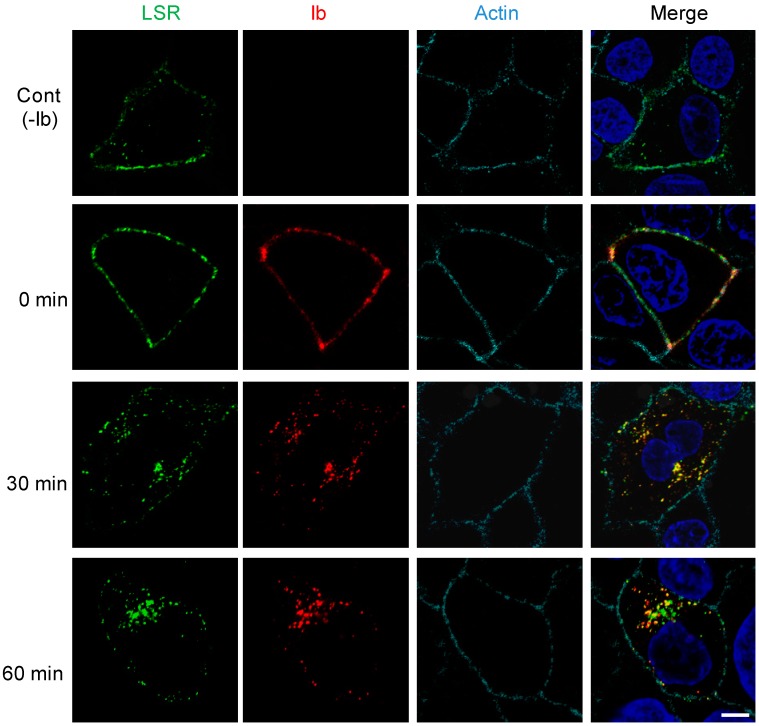
Internalization of Ib and LSR in MDCK cells. LSR-green fluorescent protein (LSR-GFP) expressing MDCK cells were treated with Ib (0.5 μg/mL) at 37 °C for the given periods. Cells were fixed, permeabilized, and stained with the anti-Ib antibody, Alexa Fluor 647-conjugated phalloidin and 4’,6’-diamino-2-phenylindole (DAPI). Ib (red), LSR (green), actin (cyan) and the nucleus (blue) were analyzed by a confocal fluorescence microscopy. A typical example from one of three experimental studies is shown, bar 5.0 μm.

We previously reported that endocytosed Ib was delivered to early endosomes [6,12]. To address the issue of whether Ib is endocytosed with LSR, we transfected MDCK cells with plasmid encoding LSR-green fluorescent protein (LSR-GFP), a fusion protein. As shown in Figure 2, LSR-GFP was mainly found in the plasma membrane in the absence of Ib. After the cells expressing LSR-GFP had been incubated with Ib at 4 °C, Ib colocalized with LSR at the plasma membrane. When shifted from 4 °C to 37 °C, Ib was endocytosed into the cytoplasmic vesicles. After 30 min, Ib coexisted with LSR in endosomes. After 60 min, Ib partially colocalized with LSR. These results demonstrated that Ib is endocytosed with LSR.

### 2.3. Clustering of LSR into Lipid Raft Microdomains by Ib

**Figure 3:**
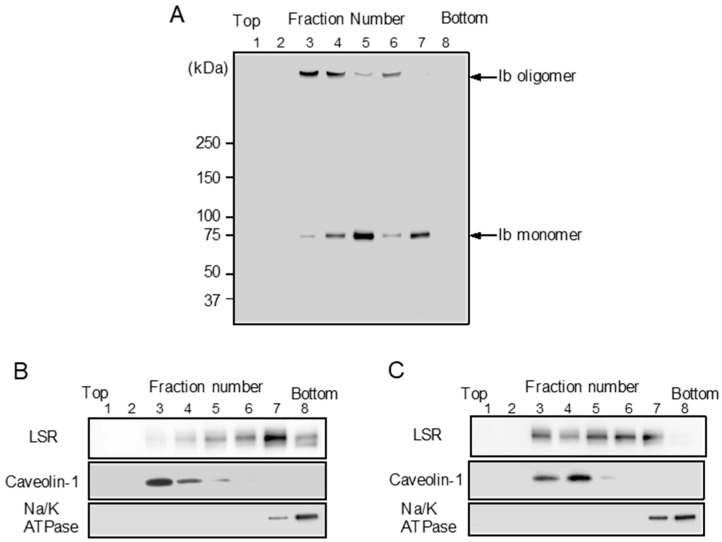
Sucrose density gradient analysis of Ib-treated MDCK cells. MDCK cells were incubated with Ib (0.5 μg/mL) (**A**,**C**) or PBS (control) (**B**) at 37 °C for 30 min. After washing, cells were lysed in 1% Triton X-100, and detergent-insoluble fractions were floated on a step sucrose gradient. Aliquots of the eight fractions from the gradient were subjected to sodium dodecyl sulfate-polyacrylamide gel electrophoresis (SDS-PAGE). Ib, LSR, a lipid raft protein (caveolin-1) or a non-lipid raft protein (Na^+^/K^+^-ATPase α1) were determined by immunoblotting. A typical example from one of three experimental studies is shown.

LSR is also the receptor for *Clostridium difficile* CDT [9]. CDT causes clustering of LSR into plasma membrane lipid rafts [25]. We reported that Ib binds to a host cell receptor in the plasma membrane and then moves to lipid rafts [11,13]. We investigated the cytoplasmic membranous distribution of LSR before and after treatment of MDCK cells with Ib. To determine the Ib binding to lipid rafts of MDCK cells, MDCK cells were treated with Ib at 37 °C, and then the cells were incubated with Triton X-100 at 4 °C for 60 min. The solubilized cells were fractionated by flotation-centrifugation. As shown in Figure 3A, Ib oligomer was wholly contained in fraction 3–4. Caveolin-1 was recovered in fraction 3–4, confirming that the fraction is enriched in lipid rafts, while Na/K ATPase alpha1, a non-raft marker protein, is detected in fraction 7–8 (Figure 3B). These data showed that Ib oligomer is located mainly within plasma membrane lipid rafts. To establish what role lipid rafts play in organizing the endocytic pathway of Ib in MDCK cells, we examined the distribution of LSR in sucrose gradient fractions. In control cells, a substantial amount of LSR was present in non-lipid raft fractions (Figure 3B). When the cells were incubated with Ib at 37 °C, LSR moved from non-lipid raft fractions to lipid raft fractions (Figure 3C). These results indicated that the binding of Ib to LSR promotes the movement of LSR into lipid rafts.

### 2.4. Interaction of N-Terminal Extracellular Region of LSR with Ib

**Figure 4:**
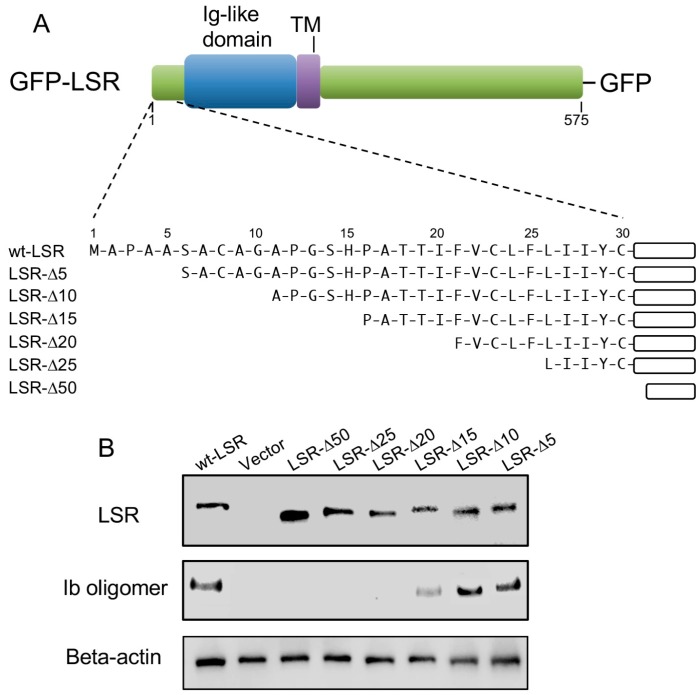
Mapping of N-terminal region of LSR responsible for the interaction of Ib. (**A**) Schematic representation of wild-type LSR or N-terminal deletion mutants of LSR-GFP fusion protein; (**B**) Wild-type LSR-GFP or LSR derivative-GFP constructs were transiently expressed in A549 cells by electroporation. The cells were incubated with Ib (0.5 μg/mL) at 37 °C for 30 min. Cell lysates were subjected to Western blot analysis using specific antibodies against Ib, LSR, and β-actin. A typical example from one of three experimental studies is shown.

LSR is a type I transmembrane protein, composed of an N-terminal region, Ig-like domain, a single transmembrane domain (TM), and a long cytoplasmic tail (Figure 4A) [16,17]. We have focused our studies on the role of the N-terminal extracellular region of LSR in the interaction of Ib. To determine the importance of the LSR amino terminus in the interaction with Ib, we constructed a series of amino-terminal truncation mutants (ΔN_5_, ΔN_10_, ΔN_15_, ΔN_20_, ΔN_25_, and ΔN_50_) fused to GFP (Figure 4A). A549 cells (which express little LSR) were transfected with a wild-type or deletion mutant of LSR-GFP. Immunoblot analysis with an anti-LSR antibody displayed similar levels of wild-type or mutant in the transfected cells (Figure 4B). Ib was added to wild-type or mutant LSR-A549 cells at 37 °C for 30 min, and bound Ib was detected by immunoblotting, utilizing an anti-Ib antibody. Unlike wild-type LSR, deletion of 15 amino acids from the extracellular N-terminus markedly inhibited the binding between the two proteins. The binding of Ib to ΔN_5_ or ΔN_10_ mutants was similar to that in wild-type LSR. Ib did not bind to ΔN_20_, ΔN_25_, or ΔN_50_ mutants. These data showed that N-terminal extracellular amino acids 10–15 of LSR is indispensable for Ib binding.

## 3. Discussion

In the present study, we demonstrated that Ib: (i) bound to LSR and formed an oligomer, (ii) induced the clustering of LSR in lipid raft membranes, (iii) internalized with LSR to endosomes, and (iv) recognized the N-terminal 10–15 amino acids of LSR. Our results showed that the binding of Ib to LSR contributes to the iota toxin-induced cytotoxicity.

Iota toxin enters sensitive cells and induces cytotoxic activity by making use of the endocytic trafficking system [1,2,3,4,5,6]. LSR has been identified as target cellular receptor for clostridium binary toxins, including Ib, CDTb and CSTb [9,15]. Ib binds to LSR on the cell surface via the C-terminal region of Ib and clusters in plasma membrane lipid rafts, and then the Ia associated with Ib oligomer configured in the rafts internalizes the target cell [6,9,12]. In this research, transfection of MDCK cells with siRNA targeting LSR impaired the binding of Ib and iota toxin-induced cytotoxicity, which validated Ib binding to LSR. Furthermore, Ib caused LSR clustering into lipid rafts. Recently, we showed that Ib accelerates ASMase release via lysosomal exocytosis and production of ceramide on the external surface of the plasma membrane [14]. Since plasma membrane ceramides have an intrinsic propensity to form clusters, ceramide spontaneously coalesces into ceramide-rich platforms in which cell-surface receptors cluster [26]. Therefore, ceramide production induced by Ib may facilitate the clustering of Ib-LSR complex into lipid rafts. Our study demonstrated that Ib is endocytosed with LSR, and co-localized to cytoplasmic vesicles. It has been reported that LSR is involved in the binding of apolipoproteins B and E, leading to their internalization [27,28,29]. We think that Ib enters the host cells using the process of LSR-mediated endocytosis. 

LSR is a type I single-pass transmembrane receptor with a surface-exposed N-terminal region [16,17,19]. The N-terminal exodomain of mouse LSR can be subclassified into two discrete parts; a poorly characterized region (~33 amino acids), followed by an Ig-like domain [16,17,19]. It has been reported that the extracellular region of LSR is needed for the binding of CDTb [30]. Our present data show that deletion of 15 amino acids from the N-terminus of LSR leads to a drastic decrease in Ib binding. Our data indicate for the first time that Ib can interact with the first 15 amino acids of LSR. The C-terminal domain of Ib (Ib421–664) is involved in LSR-binding [6,23,24]. It has been reported that LSR participates in the formation of tricellular tight junctions [19]. Recently, it was reported that Ib421-664 regulates tricellular tight junctions and elevates the intestinal absorption of large molecules via the paracellular route by disrupting tricellulin recruitment [23]. Moreover, Ib421–664 injection into mice increased the permeability of the blood-brain barrier and enabled transient delivery of subsequently administered drugs into the brain and spinal cord [24]. Thus, Ib421–664 represents an absorption enhancer specifically targeting tricellular tight junctions. Our findings demonstrate that the C-terminal domain of Ib modulates LSR via binding to its 10–15 N-terminal amino acids.

## 4. Conclusions

Our findings reveal the functional interaction of Ib with LSR. Initial binding of Ib to 10–15 N-terminal amino acids of LSR leads to clustering of the Ib-LSR complex to lipid rafts. The iota toxin-LSR complex is then endocytosed in cytoplasmic vesicles via a lipid raft-dependent pathway.

## 5. Materials and Methods 

### 5.1. Materials

Ia and Ib recombinant proteins were expressed and purified as reported previously [10,11]. Rabbit anti-Ib antibody was generated as described earlier [13]. Rabbit anti-LSR (X-25) antibodies, Na^+^/K^+^-ATPase α1 antibodies, and anti-β-actin antibodies were obtained from Santa Cruz Biotechnology (Santa Cruz, CA, USA). Mouse anti-caveolin-1 antibodies were purchased from BD Biosciences (Tokyo, Japan). Dulbecco’s modified Eagle’s medium (DMEM), Hanks’ balanced salt solution (HBSS), Alexa Fluor 568-conjugated goat anti-rabbit IgG, Alexa Fluor 647-conjugated phalloidin, and 4′,6′-diamino-2-phenylindole (DAPI) were obtained from Thermo Fisher Sci. (Tokyo, Japan). Horseradish peroxidase-labeled anti-rabbit IgG and Amersham ECL Western blotting detection reagents were obtained from GE Healthcare (Tokyo, Japan). 

### 5.2. Cell Culture and Assay of Cytotoxicity 

MDCK cells and A549 cells were purchased from the Riken Bioresource Center (Ibaraki, Japan). Cells were cultivated at 37 °C and 5% CO_2_ in DMEM containing 10% heat-inactivated fetal calf serum (FCS), 100 µg/mL of streptomycin, 100 units/mL of penicillin, and 2 mM l-glutamine (FCS-DMEM). For cytotoxicity experiments, cells were seeded into 48-well plates and incubated in FCS-DMEM, with various concentrations of Ia and Ib. After a 4-h incubation, changes in cell morphology were evaluated by microscopy, as described previously [13].

### 5.3. Plasmids and Transfection

The pCAGGS vector encoding mouse LSR-green fluorescent protein (GFP) gene was a kind gift from Professor Mikio Furuse (Kobe University, Kobe, Japan). All N-terminal deletion LSR mutants were generated by polymerase chain reaction from a pCAGGS vector, encoding mouse LSR-GFP gene as a template, and were cloned into the mammalian expression vector pCAGGS vector. Plasmid DNA was transfected into MDCK cells or A549 cells using the Neon™ transfection system (Thermo Fischer Sci., Tokyo, Japan) according to the manufacturer’s recommended protocols. After electroporation, cells were seeded into polylysine-coated glass-bottomed dishes (Matsunami Glass Co., Osaka, Japan) or 24-well plates and incubated at 37 °C and 5% CO_2_. Experiments were performed within 24 h after electroporation with the plasmid DNA.

### 5.4. Western Blotting 

Cell lysates, prepared in SDS sample buffer, were subjected to sodium dodecyl sulfate-polyacrylamide gel electrophoresis (SDS-PAGE), followed by Western blotting analysis using specific antibodies as previously described [13].

### 5.5. RNAi-Mediated Suppression of LSR 

siRNAs for LSR and siRNA negative controls were purchased from Qiagen (Tokyo, Japan). In knockdown experiments, all transfection assays were done using a Neon™ transfection system following the manufacturer’s protocol (Thermo Fischer Sci.). Mixed solutions of MDCK cells (2 × 10^6^ cells) and siRNA (500 pmol) were electroporated. Transfected cells were assayed 48 h after transfection [14].

### 5.6. Immunofluorescence Microscopy 

For immunofluorescence microscopy of cultured cells, cells were fixed in 3% paraformaldehyde in 0.1 M phosphate buffer (pH 7.3) for 10 min at room temperature after the treatment with Ib. Paraformaldehyde-fixed cells were quenched by 20 mM NH_4_Cl for 10 min, and permeabilized with 0.1% Triton X-100 in PBS for 15 min at room temperature. After washing with PBS, cells were blocked with 2% bovine serum albumin in PBS for 1 h, and incubated with rabbit anti-Ib antibody in PBS containing 2% BSA at room temperature for 2 h, followed by incubation with Alexa Fluor 568-conjugated anti-rabbit IgG in PBS containing 2% BSA at room temperature for 2 h. The nuclei were stained with DNA dye DAPI for 30 min [12]. For actin staining, cells were incubated with Alexa Fluor 647-conjugated phalloidin for 2 h. Fixed samples were analyzed with a Nikon A1 laser scanning confocal microscope (Tokyo, Japan).

### 5.7. Detergent-Resistant Membrane Isolation 

Detergent-resistant membranes were isolated as described previously [13]. Briefly, MDCK cells grown on 100 mm diameter dishes were treated with Ib. Cells were then lysed in 1% Triron X-100 at 4 °C for 30 min in Hanks balanced salt solution (HBSS) containing the protease inhibitor cocktails. The lysate was mixed with an equal volume of 80% sucrose (*w*/*v* in HBSS), and a step gradient was prepared by overlaying with 30% sucrose in HBSS followed by a last step of 5% sucrose. The gradient was centrifuged at 45,000 rpm (250,000× *g*) for 18 h at 4 °C using a Beckman SW55 rotor. Eight fractions were collected from the top of the gradient, and an aliquot of each was mixed with an equal volume of 2× SDS-sample buffer, boiled for 3 min, and evaluated by SDS-PAGE and Western blotting. 

### 5.8. Statistical Analysis

Statistical tests were assessed by Easy R (Saitama Medical Center, Jichi Medical University, Shimotsuke, Japan) [31]. Differences between two groups were examined using the two-tailed Student’s *t*-test. One-way analysis of variance (ANOVA) followed by the Tukey test was used to evaluate differences among three or more groups. A P value of less than 0.01 was considered significant.
